# Rabbit thyroid extracellular matrix as a 3D bioscaffold for thyroid bioengineering: a preliminary in vitro study

**DOI:** 10.1186/s12938-021-00856-w

**Published:** 2021-02-09

**Authors:** Jie Weng, Bi Chen, Mengying Xie, Xinlong Wan, Peng Wang, Xiaoming Zhou, Zhiliang Zhou, Jin Mei, Liang Wang, Duping Huang, Zhibin Wang, Zhiyi Wang, Chan Chen

**Affiliations:** 1 Department of Emergency Medicine and General Practice, The Second Affiliated Hospital and Yuying Children’s Hospital of Wenzhou Medical University, Wenzhou, 325027 China; 2 Department of Surgical Oncology, Wenzhou People’s Hospital, The Wenzhou Third Clinical Institute Affiliated With Wenzhou Medical University, Wenzhou, 325000 China; 3 Department of Geriatric Medicine, The First Affiliated Hospital, Wenzhou Medical University, Wenzhou, 325000 China; 4 Institute of Bioscaffold Transplantation and Immunology, School of Basic Medical Sciences, Wenzhou Medical University, Wenzhou, 325035 China; 5 Department of Public Health, Robbins College of Health and Human Sciences, Baylor University, Waco, TX USA; 6 Department of Thyroid and Breast Surgery, The First Affiliated Hospital of Wenzhou Medical University, Wenzhou, 325000 China; 7 Center for Health Assessment, Wenzhou Medical University, Wenzhou, China

**Keywords:** Thyroid gland, Decellularization, Extracellular matrix, Scaffold, Organ engineering

## Abstract

**Background:**

Advances in regenerative medicine technologies have been strongly proposed in the management of thyroid diseases. Mechanistically, the adoption of thyroid bioengineering requires a scaffold that shares a similar three-dimensional (3D) space structure, biomechanical properties, protein component, and cytokines to the native extracellular matrix (ECM).

**Methods:**

24 male New Zealand white rabbits were used in this experimental study. The rabbit thyroid glands were decellularized by immersion/agitation decellularization protocol. The 3D thyroid decellularization scaffolds were tested with histological and immunostaining analyses, scanning electron microscopy, DNA quantification, mechanical properties test, cytokine assay and cytotoxicity assays. Meanwhile, the decellularization scaffold were seeded with human thyroid follicular cells, cell proliferation and thyroid peroxidase were determined to explore the biocompatibility in vitro.

**Results:**

Notably, through the imaging studies, it was distinctly evident that our protocol intervention minimized cellular materials and maintained the 3D spatial structure, biomechanical properties, ECM composition, and biologic cytokine. Consequently, the decellularization scaffold was seeded with human thyroid follicular cells, thus strongly revealing its potential in reinforcing cell adhesion, proliferation, and preserve important protein expression.

**Conclusions:**

The adoption of our protocol to generate a decellularized thyroid scaffold can potentially be utilized in transplantation to manage thyroid diseases through thyroid bioengineering.

## Background

Several diseases have been associated with the thyroid gland and causes various medical conditions. These diseases can be either congenital, such as congenital hypothyroidism (pediatric condition), that affects the intelligence development and impairs physical growth, or be an acquired condition, such as thyroid cancer, surgery, and trauma [1, 2]. However, the main treatments for hypothyroidism are hormone replacement therapy (HRT) and thyroid gland transplantation [3, 4]. Moreover, thyroid hormone overdose or deficiency can be the major problem of HRT with the possibility of inducing cardiovascular diseases. Associated and critical limitations with thyroid gland transplantation are lack of donor organs, chronic immune rejection, and lasting immune suppression [5, 6]. Hence, the adoption of tissue engineering (TE) techniques may provide a promising therapeutic alternative that can create viable biomaterials for the thyroid gland.

Developing biomaterials that integrate with the 3D biocompatible scaffolds, cells, and growth factors form the core of TE techniques. Besides, the preparation of 3D biocompatible scaffolds has proved to be the major challenge. For example, scaffolds, such as extracellular matrix (ECM) materials, are considered of low immune response but highly biocompatible. The decellularization technique has been reported as an important and efficient method in the production of biocompatible bio-scaffolds. The ECM materials can provide the microenvironment for cell adhesion, cell proliferation, and cellular differentiation [7, 8]. Also, the ECM has been revealed to potentially release growth factors and cytokines which can induce and regulate growth, proliferation, and differentiation of cells [9]. Decellularized scaffolds have been widely utilized for TE, such as blood vessels [10], urinary bladder [11], and skin[12], in clinical applications. The use of scaffolds has contributed to tissue reconstruction and regeneration [13, 14]. However, up to date, accepted standardized thyroid decellularization protocol is lacking.

Herein, the adoption of decellularization protocols aimed to minimize cell components and preserve 3D extracellular matrix in decellularization methods, such as physical, chemical, or enzymatic methods, with the least impact on the scaffold composition, biological activity, and mechanical integrity. Nevertheless, different tissues or organs may need to use decellularization protocols that vary in decellularization agents, however, due to their specificity, none of the methods is suitable for all decellularization protocols. For instance, Ott et al. (2008) [15] in their study, applied vascular perfusion to decellularize intact rat hearts where sodium dodecyl sulfate (SDS) and t-octylphenoxypolyethoxyethanol (Triton X-100) were involved in the decellularization protocol. Both SDS and Triton X-100 have been commonly used as detergents for cell lysis applications in TE. Notably, studies have demonstrated that perfusion decellularization protocols have been commonly performed on relatively large organs with distinct blood vessels or catheters, such as kidneys and lungs [14, 16]. More so, in tissues where blood vessels hardly separate like in small intestine submucosa (SIS) and testis tissues, immersion/agitation decellularization could be an alternative option [17, 18]. Decellularization protocols of the thyroid gland are currently limited. Therefore, in our study, we selected the immersion/agitation decellularization protocols to decellularize the thyroid gland and evaluated the decellularization properties, as well as explored the biocompatibility in vitro.

## Results

### Decellularized thyroid gland scaffolds evaluations

**Fig. 1:**
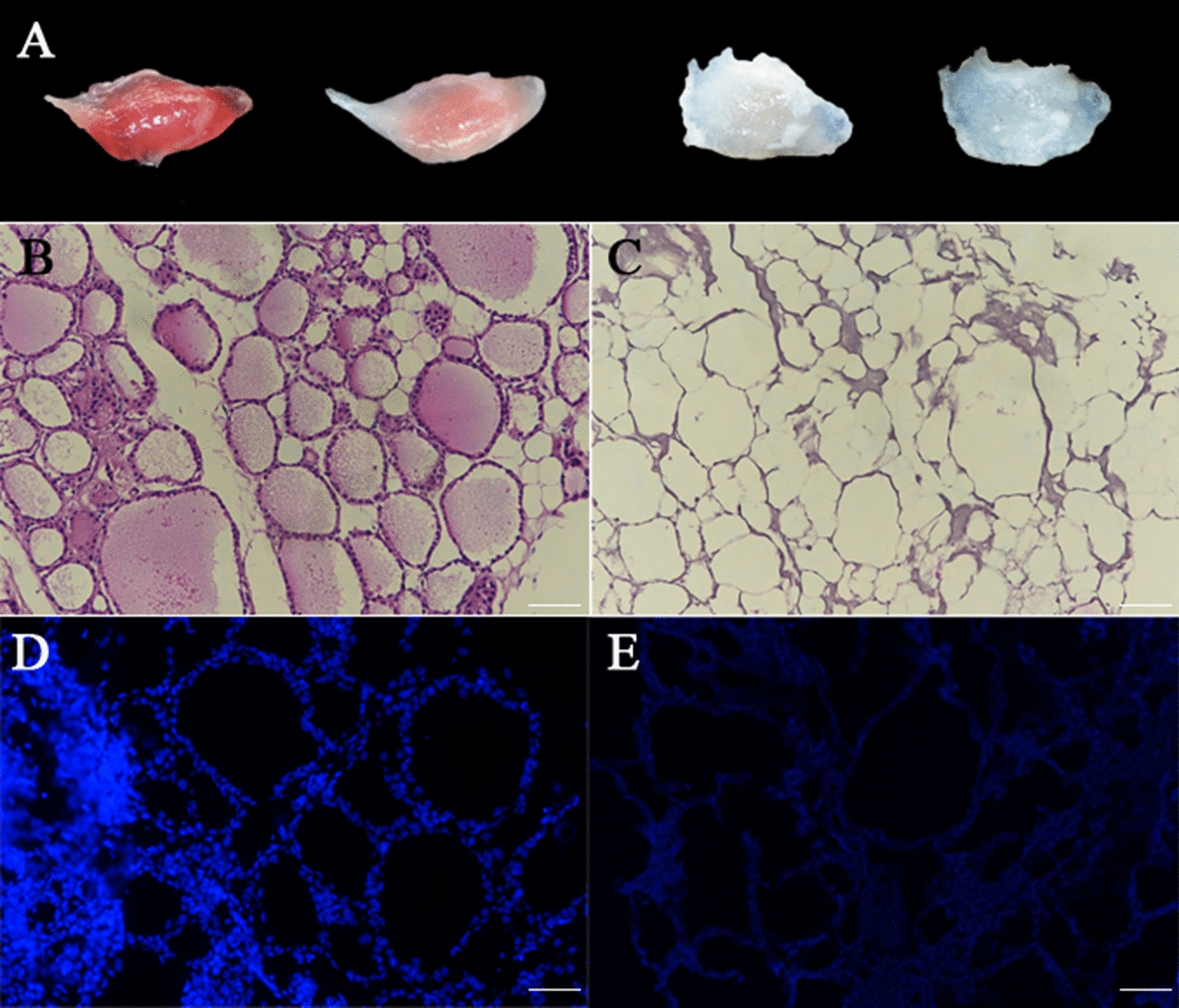
Characterization of the DTG scaffolds. Gross appearance of DTG scaffolds (**a**). H&E staining revealed the massive blue-stained cell nucleus in native thyroid gland tissues (**b**) and no visible cell nucleus in DTG scaffolds (**c**). DAPI staining revealed the existence of blue-stained nuclei in native thyroid gland tissues (**d**) but not DTG scaffolds (**e**). Scale bar = 50 µm

During the agitation decellularization process, a gradual change in the color of native thyroid gland tissue from their initial pale pink to white then became transparent. After the decellularization process, the thyroid gland became milky white translucent (Fig. 1a).

In our study, results from staining analysis by H&E revealed massive cell nucleus in native thyroid gland tissues whereas none in DTG scaffolds (Fig. 1b, c). Moreover, DAPI staining was similar to H&E staining (Fig. 1d, e).

From our extraction and quantification of the DNAs, it distinctly showed that DNA concentration in DTG scaffolds was significantly lower than the native thyroid gland (native thyroid gland = 342.41 ± 32.28, DTG scaffolds = 23.3 ± 4.41). The DNA clearance efficiency was 93%.

**Fig. 2:**
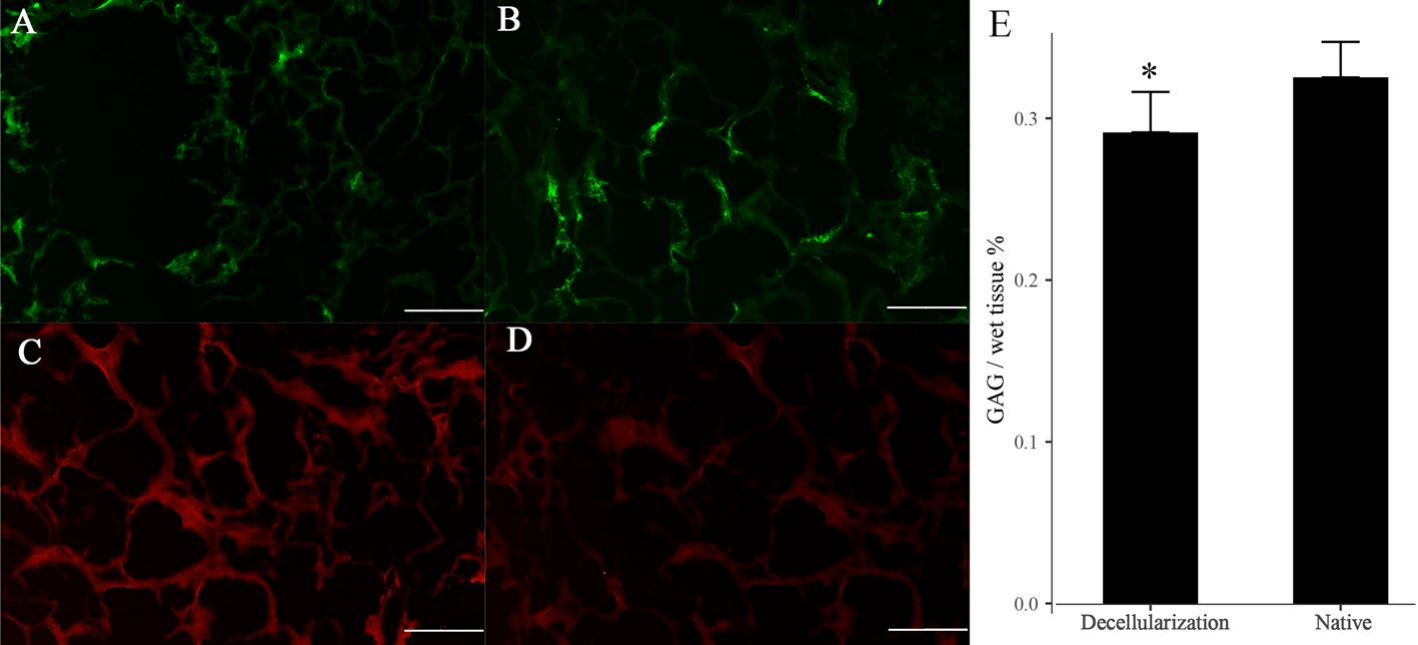
ECM characterization of the DTG scaffolds. Immunostaining showed the presence of major ECM composition–Collagen I (**a**), Collagen IV (**b**), LN (**c**), and FN (**d**) were all conserved in DTG scaffolds. Scale bar = 50 µm. Glycosaminoglycan (GAG) content (**e**) of DTG scaffolds and native thyroid. Data are shown as mean ± SD. *p < 0.05 compared to native

According to immunostaining assessments, it demonstrated that protein Collagen I and IV, LN, and FN were retained in DTG scaffolds, as well as the scaffolds' structures (Fig. 2a–d). In contrast, the content of GAG declined significantly in the DTG scaffolds as demonstrated by GAG quantification (P = 0.027; Fig. 2e).

**Fig. 3:**
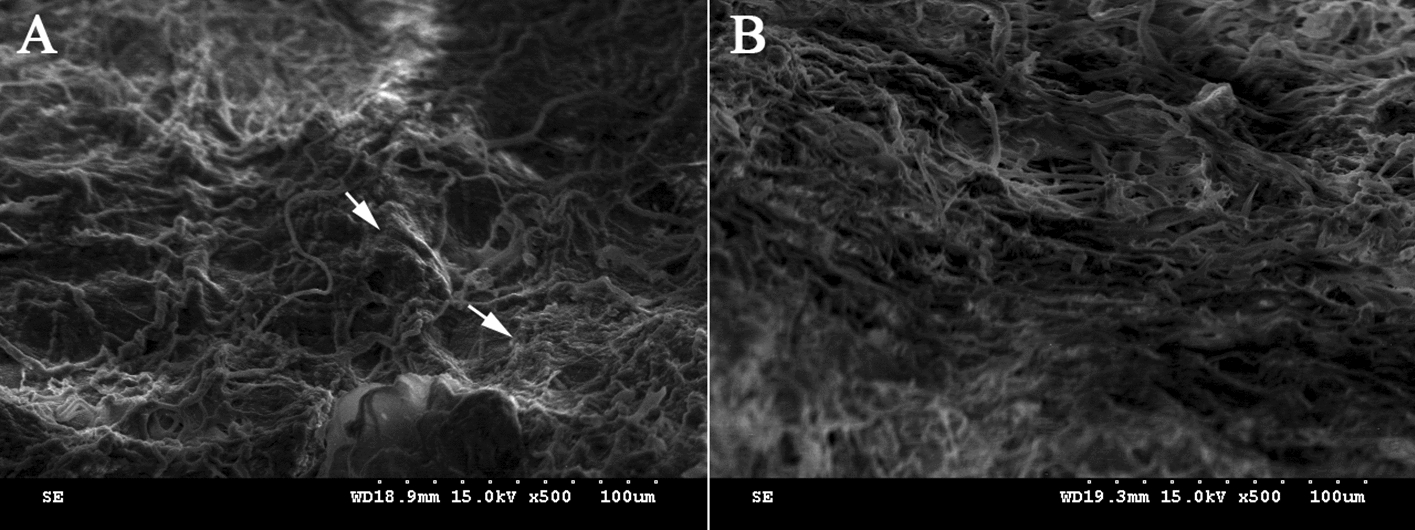
SEM images of the DTG scaffolds. SEM comparison of native (**a**) and DTG scaffolds (**b**) demonstrated preservation of 3D microstructure of thyroid gland after decellularization. The arrows in **a** represented the cells

Notably, there was no significant difference in SEM of the DTG scaffolds as compared to that of the native thyroid gland. However, the fibrous structure of DTG scaffolds was visible and preserved (Fig. 3).

**Fig. 4:**
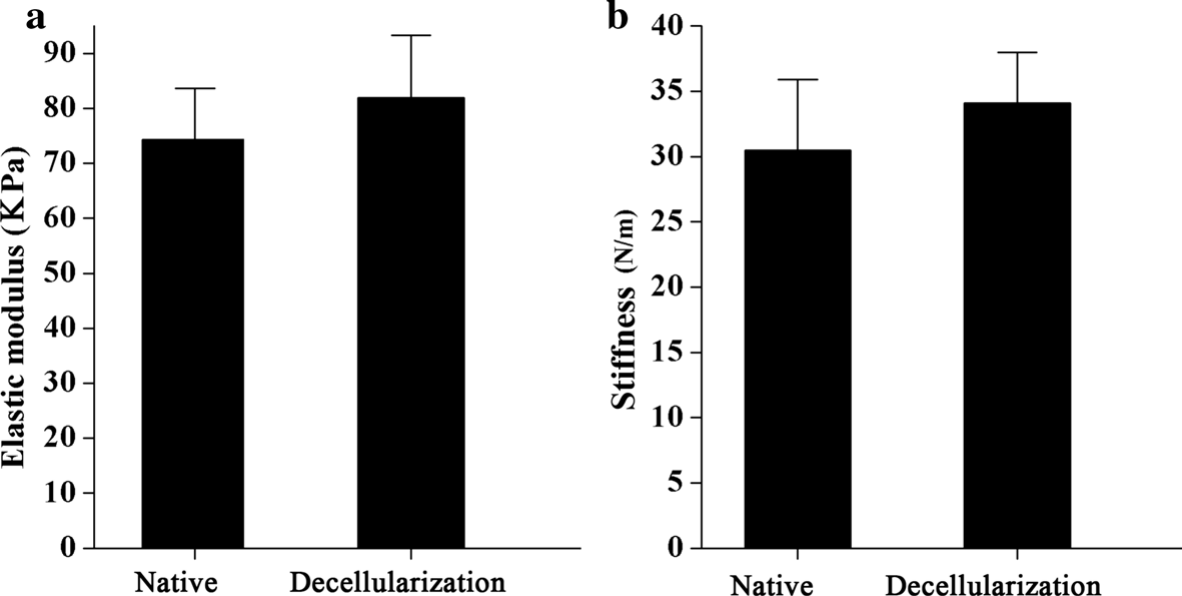
Biomechanical properties of the DTG scaffolds. Biomechanical properties comparison of native and DTG scaffolds demonstrated the elastic modulus (**a**) and stiffness (**b**) were retained after decellularization. Data are shown as mean ± SD

Here, the results of mechanical testing revealed that the mechanical characteristics of DTG scaffolds in term of toughness and elastic modulus has not been affected (K (N/m): native = 30.5 ± 5.4, decellularization = 34.1 ± 3.9, P > 0.05; E (kPa): native = 74.3 ± 9.3, DTG scaffold = 82.0 ± 11.3, P > 0.05). Thus, the native toughness and elastic modulus were retained after decellularization (Fig. 4).

**Fig. 5:**
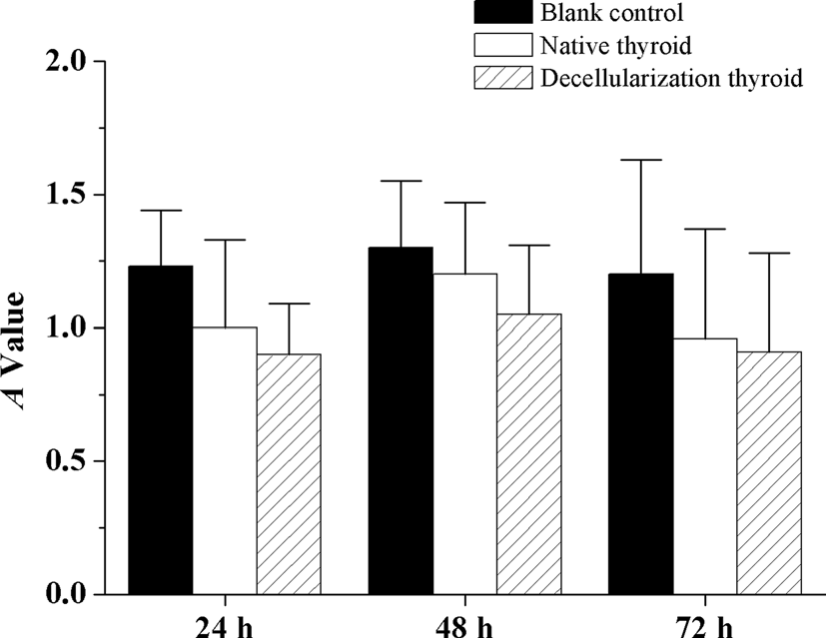
Cytotoxicity assays of the DTG scaffolds. The absorption value of the DTG scaffolds at 24 (**a**), 48 (**b**) and 72 h (**c**) is the same with the native thyroid gland. Data are shown as mean ± SD

The in vitro cytotoxicity assay of the control group and DTG scaffold groups were performed by CCK-8 assay with HTFCs. As a result, the absorption value at 24, 48, and 72 h did not show significant difference between DTG scaffolds and the native thyroid gland (Fig. 5).

### Decellularized thyroid gland scaffold biological functions evaluation

**Fig. 6:**
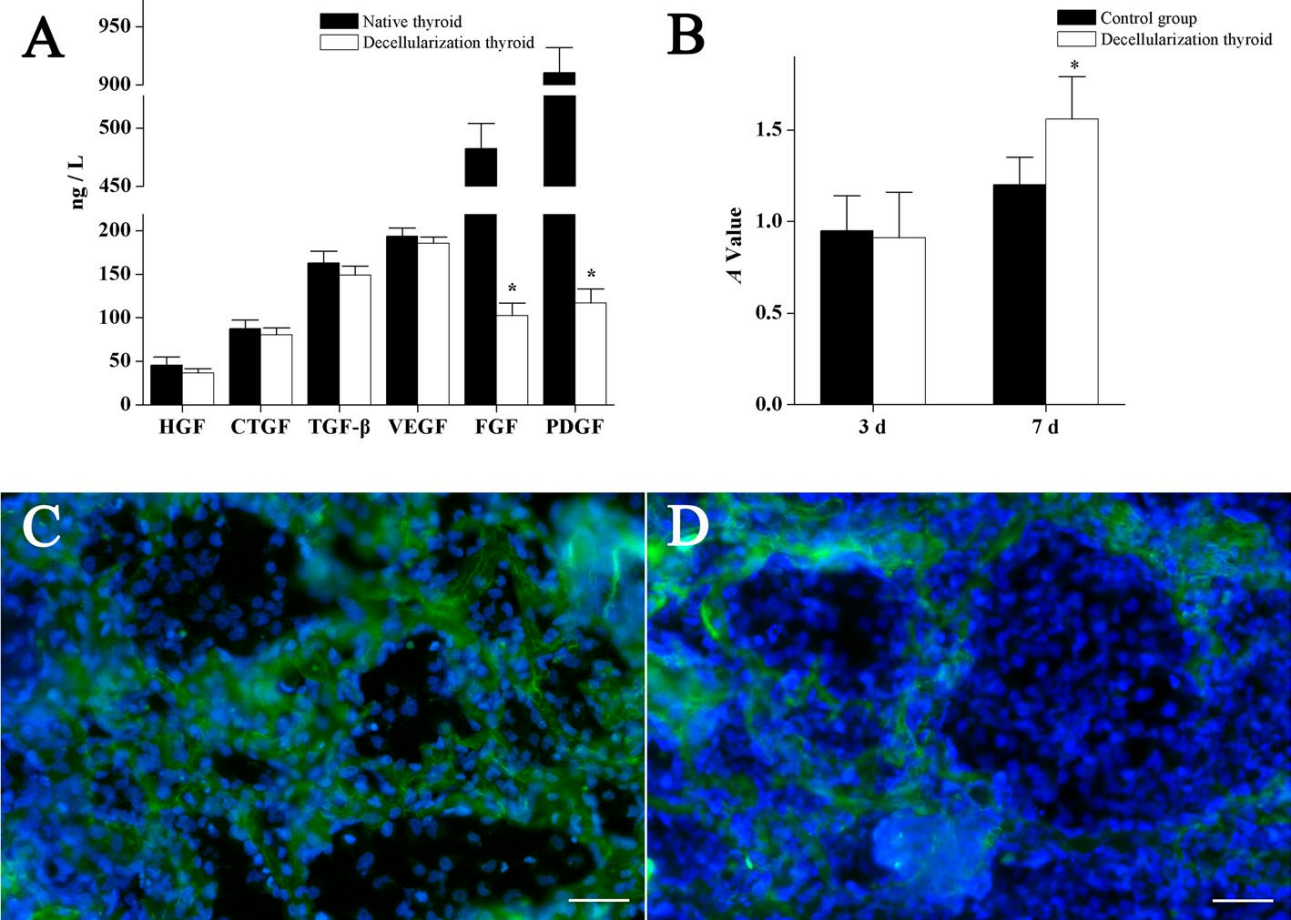
Decellularized thyroid gland scaffold biological function evaluation. Quantitative assay of cytokines in the DTG scaffolds (**a**). Effect of DTG scaffolds on cell proliferative activity (**b**). Recellularization of the DTG scaffolds (**c**, **d**). The DTG scaffolds were seeded with HTFCs to generate a co-culture system at 3 days (**c**) and 7 days (**d**). Immunostaining showed the TPO weak expression at 3 days and significantly reduced at 7 day. Data are shown as mean ± SD.*p < 0.05. Scale bar = 50 µm

The ELISA assay for quantitative analysis of cytokines (VEGF, TGF-β, HGF, FGF, CTGF, and PDGF) was performed to assess the DTG scaffolds for thyroid gland regeneration. Here, the levels of VEGF, TGF-β, HGF, and CTGF in DTG scaffolds were similar to those in native thyroid (all P > 0.05). Besides, the FGF and PDGF levels were lower in DTG scaffolds than native thyroid (both P < 0.001) (Fig. 6a).

Further assessments on cell proliferation of HTFCs in DTG scaffolds were determined. Hence, our findings showed that the cell proliferative activity in 3D scaffolds was notably increased when incubated for 7 days as compared to conventional cell culture (P = 0.012) (Fig. 6b). Also, stereological cell counting indicated that the cells at 7th day present higher density compared to the 3rd day (57,833 ± 8975 vs. 22,500 ± 8215 cells/mm^3^, respectively, P < 0.001) (Fig. 6c, d). Furthermore, immunostaining demonstrated the expression of TPO in HTFCs after 3 days, thus indicating the preservation of important cell functions when HTFCs were seeded on 3D scaffolds. However, 7 days post-seeding, the TPO expression was not observed (Fig. 6c, d).

## Discussion

In recent years, physical, chemical, and enzymatic methods have been considered as the most common approaches to discern tissue decellularization [19]. The combined use of the above methods yields the best decellularization ECM [20, 21]. As of our study, following the basic rules of decellularization, we developed a protocol for the generation of cellular biocompatibility decellularized matrix from the thyroid gland with similar characteristics to native ECM properties as an important material for thyroid tissue engineering.

Information on thyroid decellularization protocol is limited. Though, the first attempt was by Pan et al. [22], who prepared the rat decellularized thyroid ECM by perfusion through the common carotid artery. The isolating and perfusing the common carotid artery in rats was challenging, therefore, making their protocol difficult to adapt to other researchers. In our study, we exposed rabbit thyroid gland tissue to ionic detergents (SDS), as well as to mechanical agitation, and chose the most appropriate solution protocol to prepare ECM. In our previous experiment, we compared the decellularization efficiency of SDS and non-ionic detergent Triton X-100, and determined the SDS as the most effective in the decellularization of rabbit thyroid gland tissue (data not shown). Besides, studies have reported better decellularization outcomes with SDS than Triton X-100 [23, 24]. Thus, in this study, SDS was used as the cell removal agent. Immersion/Agitation for 72 h in 1% SDS can effective removal of cellular nuclei and cell components in the thyroid gland tissue. Also, the effects of different agitation frequency (50 rpm, 100 rpm, and 150 rpm) on cell removal were assessed. It was found that cells could not be adequately removed at 50 rpm, thus the degree of ECM destruction was increased to 150 rpm (data not shown). We revealed that agitation frequency at 100 rpm was the optimum condition for the decellularization of the thyroid which was consistent with the agitation frequency in Khosroshahi et al. study [25]. Collectively, this protocol not only removes DNA in thyroid gland tissue but also preserve the 3D spatial structure of the native thyroid gland.

The aims for successful decellularization are to clear cellular and the retention of ECM components such as a biologic cytokine, biomechanical properties, and 3D spatial structure [26]. However, the DTG scaffolds from our protocol achieved the stringent criteria: they lack histologically visible cellular material (DAPI or H&E staining) and with under 50 (ng/mg dry tissue) concentration of the dsDNA [27]. Since the residual DNA fragments in decellularization ECM are directly correlated to immunological rejection response upon implantation [28], therefore, the attained criteria are paramount. The DAPI and H&E staining showed no visible cellular nuclei in DTG scaffolds. Besides, the DNA quantification showed significantly low concentration than the above criteria, however, the DNA removal rate was 93%. Furthermore, the decellularization efficiency in our study was consistent with previous studies with accepted standards for organ decellularization [29, 30].

Notably, maximum preservation of ECM composition and cytokines are important for organ regeneration [31, 32]. Thus, in our study, the immunofluorescence staining demonstrated the main ECM proteins in DTG scaffolds were retained, including LN, FN, Collagen type I, and IV, which are consistent with Pan et al. [22] findings. Studies have reported that Collagens are responsible for maintaining the ECM structure [33]. Although it is widely considered decellularization with SDS is related to ECM ultrastructure disruption [28, 34], our SEM results demonstrated that the 3D ECM structures were preserved thus indicating that 1% SDS was safe to DTG scaffolds. Besides, GAGs were preserved in DTG scaffolds. It has been reported that fibronectin promotes promote cell adhesion and migration together with GAGs, indicating the importance of GAGs in scaffold function [35, 36]. Although the rabbit thyroid gland tissues were completely decellularized, the important cytokines (VEGF, TGF-β, HGF, and CTGF) were preserved in the DTG scaffolds which was similar to the kidney decellularized scaffold [14]. The cytokines together with FN and GAGs, maybe contributing to cell growth and adhesion when the HTFCs are seeded into scaffolds.

Production of decellularized scaffolds that approximates biomechanical properties of native ECM is vital in organ bioengineering, as it contributes to maintaining the structural integrity of scaffolds after transplantation and appropriate cell–matrix interaction [37]. Studies have demonstrated that changes in ECM biomechanical properties occur in varying degrees after decellularization [38]. Therefore, we purposely evaluated biomechanical properties of the decellularized thyroid gland and from the ECM findings, it was evident that the elastic modulus and toughness properties showed similarity to native thyroid gland tissue, which was similar with others studies [39–41]. As of this, it can be attributed that elastin and collagen content of DTG scaffolds were preserved. Although the impact of DTG scaffolds mechanical properties on thyroid gland cell function has not been further elucidated in this study, it has great significance for decellularization thyroid transplantation in the future.

Thyroid structure reconstruction and endocrine function restoration are the ultimate goals in thyroid organ bioengineering and regeneration. Numerous studies have explored methods to reconstruct thyroid gland tissues [42–44]. Toda et al. [45] were the first researchers to reconstruct the thyroid follicles in three-dimensional collagen gel (main component is acid-soluble type I collagen) in an in vitro experiment. Besides, Toni et al. [46] rebuilt the stromal/vascular scaffolds of the human thyroid gland via in vivo visualization and computer techniques. Synthetic scaffolds can meet some biological engineering requirements, but may lack complete native ECM components and bioactive factors, which are specifically required for thyroid gland cell attachment, migration, proliferation. Therefore, decellularization scaffolds seem to provide an attractive strategy to satisfy the organ bioengineering needs. Native thyroid decellularization ECM scaffolds have the potential to offer proper microenvironment for thyroid gland cells. Despite recellularization ECM materials implemented in some organs, the development of thyroid gland bioengineering significantly later than other organs, as only pan et al. reported successful recellularization of native thyroid gland ECM so far [15]. In addressing the recellularization of the DTG scaffolds issue, in our study, two steps were adopted. In the first step, we explored whether DTG scaffolds displayed toxicity to HTFCs. Due to the decellularization detergent SDS used in our study, it may be toxic to host cells when the DTG scaffolds are implanted. However, the scaffolds expressed no effect to proliferative activity when HTFCs were exposed to DTG scaffolds, it is indicative the residual SDS was successfully removed or was below the safe level [47]. On the second step, thyroid follicular cells were seeded onto DTG scaffolds to assess the ability of cell adhesion and proliferation in vitro. The HTFCs adhered to the DTG scaffolds and subsequently infiltrate deeper into the scaffolds where the cells preserved the TPO expression 3 days post-seeding. The TPO expression declined after 1 week, probably due to deficiency in essential hormones, such as thyroxin stimulating hormone. Besides, DTG scaffolds can improve proliferation which was detected by CCK-8. This is likely due to the reason that DTG scaffolds retained the VEGF, TGF-β, HGF, and CTGF important cytokines and 3D environment that contributed to cell proliferation. Previous studies have demonstrated that cell–matrix interaction in 3D scaffolds are more favorable from those on 2D environment [48, 49]. These results illustrate that seeded cells and DTG scaffolds can maintain well biocompatibility in vitro. Also, these characteristics contributed to thyroid gland regeneration which is crucial in organ bioengineering.

In our study, we have demonstrated the DTG scaffolds with characteristics of native thyroid, have good biocompatibility in vitro, and can promote thyroid cell proliferation with important significance in thyroid organ bioengineering and regeneration. We have explored its properties preliminary in vitro, and we will implant the scaffold cell co-culture system in vivo and examine thyroid hormone expression next.

## Conclusions

In our study, we successfully developed a thyroid gland decellularization protocol that uses the immersion/agitation method. However, thorough characterization, it demonstrated that the DTG scaffolds preserved native 3D spatial structure, biomechanical properties, important ECM composition, and cytokines. Moreover, the DTG scaffolds exhibited good cytocompatibility, support HTFCs growth, and proliferation. Therefore, a decellularization scaffold is likely to serve as a platform for thyroid regeneration and transplant.

## Materials and methods

### Animals

In this experimental study, 24 male New Zealand white rabbits, weighing 3–4 kg, were sourced from Shanghai SLAC Laboratory Animal Co., Ltd. Besides, the rabbits were housed in a room maintained on a 12/12 h light/dark cycle and continuously supplied with food and water. Also, in our study, the use of animals was approved by the Animal Studies Ethics Committee of the Second Affiliated Hospital of Wenzhou Medical University.

### Immersion/agitation decellularization protocol

Briefly, the New Zealand white rabbits were anesthetized using intraperitoneal injection with 6% sodium pentobarbital (0.5 ml/kg). Thereafter, heparinization of the rabbits was performed by injecting heparin (3 mg/kg) through the marginal ear vein. The anterior neck muscles were opened to exposed, separated, and removed the envelope surrounding the thyroid gland. The thyroid gland was excised and placed in a phosphate buffer saline (PBS) glass container. Samples collected from the thyroid gland were placed in the Petri dish and mechanically agitated by the shaker before their complete immersion in 1% (v/v) SDS. Hence, decellularization in 1% SDS was administered for 72 h, at a frequency of 100 rpm following its replenished after every 4 h. After the decellularization, thyroid gland scaffolds were washed in deionized water for another 24 h. Replacement of the deionized water was done every 4 h. However, the thyroid gland scaffolds were stored at 4℃ in PBS with 1% (v/v) 100 U/ml penicillin and 100 μg/ml streptomycin (P/S) for subsequent applications.

### Histological and immunostaining analyses

Fresh native and decellularized thyroid gland (DTG) scaffolds were fixed in 4% paraformaldehyde and sliced to 5 μm-thick sections. For tissue structure analysis, hematoxylin and eosin (H&E) were performed on deparaffinized sections, where images were captured by a light microscope.

Besides, the sections were operated according to the manufacturer's procedures for immunostaining analysis. Here, the collected samples were incubated with primary antibodies against collagen I (1:100, Invitrogen), collagen IV (1:100, Invitrogen), laminin (LN) (1:100, Invitrogen) and fibronectin (FN) (1:100, Invitrogen) at 4℃ overnight. Afterwards, the samples were incubated with secondary antibodies (DyLight 488- or 594-) (1:200, Santa Cruz). Besides, the cell nuclei were stained with 4, 6-diamidino-2-phenylindole (DAPI) (1:10,000, Beyotime) after being rinsed with PBS. Consequently, immunostaining images were taken by a fluorescence microscope (Nikon, Japan).

### Glycosaminoglycan (GAG) quantification

The sulfated GAG content of native and DTG scaffolds was quantified using the Blyscan GAG Assay Kit (Biocolor Life Sciences, Carrickfergus, UK). In brief, 50 mg of minced wet tissue was weighed and placed in a centrifuge tube containing 0.1 mg/ml proteinase K (Sigma) at 50 °C for 24 h with gentle agitation. Aliquots of each sample were mixed with 1,9-dimethyl-methylene blue dye and reagents from the GAG assay kit. The absorbance was assayed with a microplate reader at 450 nm.

### Scanning electron microscopy (SEM)

Fresh native and DTG scaffolds were fixed in 10% formalin buffer at 4℃ overnight after extensively washed in deionized water. The fixed tissues were dehydrated in graded ethanol solutions after rinsing. Thereafter, dehydrated tissues were soaked in liquid carbon dioxide for critical point drying, sliced into 1 mm thickness sections, and analyzed by SEM (S3000-N, Hitachi).

### DNA quantification

The extraction of total DNA was done using the TRIzol reagent (TaKaRa Inc., Kyoto, Japan) from fresh native and DTG scaffolds. The DNA was quantified using ultraviolet spectrophotometry (OnedropTM OD-1000, PerkinElme).

### Mechanical properties test

Mechanical properties were carried out using a mechanical analyzer (Zwick/Roell, BZ2.5/TN1S, Germany). The tissue specimens were subjected to uniaxial tension until failure. The fresh native and DTG scaffolds were cut into 5*5 mm^2^ pieces before rehydrated with PBS for 1 h. The specimens were mounted on the Instron, a preloading of 0.015 N was imposed and the specimens length were reported, then a preload of 0.003 N was set. The elastic modulus and stiffness were calculated to evaluate the mechanical properties.

### Cytokine assay

The enzyme-linked immunosorbent assay (ELISA) kit (R&D Systems, USA) was used to analyze cytokines in DTG scaffolds. The concentrations of various cytokines including vascular endothelial growth factor (VEGF), transforming growth factor-β(TGF-β), hepatocyte growth factor (HGF), fibroblast growth factor (FGF), connective tissue growth factor (CTGF) and platelet-derived growth factor (PDGF) were assayed with a microplate reader at 450 nm.

### Cell culture

Human thyroid follicular cells line (HTFCs) were purchased from the Chinese Academy of Sciences Kunming Cell Bank (Kunming, Yunnan, China). The HTFCs were cultured in Dulbecco’s modified Eagle’s medium (Gibco, Grand Island, USA) supplemented with 10% fetal bovine serum (FBS) (Invitrogen, Carlsbad, CA), 1% (v/v) penicillin and streptomycin under cell incubation conditions of 37ºC with 5% CO_2_ air atmosphere.

### Cytotoxicity assays

The cytotoxicity of DTG scaffolds was conducted through the Cell Counting Kit-8 (CCK-8) assay. Herein, decellularized samples were sliced into 1 mm thickness sections before sterilized adequately with P/S. After placing the sections at the bottom of the 96-well plate, they were seeded with HTFCs (1 × 10^5^ cells per well) for 24, 48, 72 h at 37ºC with 5% CO_2_ complete medium. Experimentally, HTFCs without exposure to any samples were used as control. Moreover, six wells per sample were prepared and the tests were triplicated.

### Recellularization of decellularized scaffolds

Accordingly, the DTG scaffolds were sliced into sections of 0.5 mm thickness and 5 mm in diameter (3D scaffolds) following sterilizing with P/S before seeding. Thereafter, the sections were placed at the bottom of the six-well plates, followed by seeding with HTFCs at a density of 1 × 107 cells per well. Then, the seeded scaffolds were cultured in a cell incubator (37°C, 5% CO_2_) with a complete medium for 7 days. The proliferation of HTFCs on DTG scaffolds was examined by CCK-8. Besides, immunostaining analysis was conducted to detect the thyroid peroxidase (TPO) expression of HTFCs in DTG scaffolds.

### Statistical analysis

Data were represented as the mean ± standard deviation (SD). Student's t test was adopted in two groups, whereas a one-way analysis of variance was used for the multi-groups (SPSS statistics software v.20, IBM). The level of significance was set at *p* = 0.05.

## Data Availability

Data sharing not applicable to this article as no datasets were generated or analyzed during the current study.
